# Disparities in Delivery of Ambulatory Surgical Care for Children

**DOI:** 10.1001/jamanetworkopen.2023.17018

**Published:** 2023-06-05

**Authors:** Yao Tian, Lindsay D. Allen, Martha-Conley E. Ingram, Mehul V. Raval

**Affiliations:** 1 Department of Surgery, Feinberg School of Medicine, Northwestern University, Chicago, Illinois; 2 Department of Emergency Medicine, Feinberg School of Medicine, Northwestern University, Chicago, Illinois; 3 Division of Pediatric Surgery, Department of Surgery, Northwestern University Feinberg School of Medicine, Ann & Robert H. Lurie Children’s Hospital of Chicago, Chicago, Illinois

## Abstract

This cross-sectional study characterizes the delivery of ambulatory surgical care for children across freestanding ambulatory surgery centers and hospital-based outpatient centers and tests for differences in patient characteristics and features of procedures being performed.

## Introduction

More than half of surgical procedures are delivered in hospital-based outpatient centers (HBOCs) or freestanding ambulatory surgery centers (FASCs); the latter are owned primarily by physicians.^1,2^ Although FASCs are thought to offer lower-cost and more efficient surgical care compared with HBOCs,^3^ studies of adult patients show that FASCs may selectively choose patients in need of more profitable procedures, or with more generous insurance plans.^4,5^ In the US, nearly 40% of children are covered by public insurance (ie, Medicaid and Children’s Health Insurance Program [CHIP]), and, therefore, selectively choosing patients may disproportionally affect pediatric surgical patients. This cross-sectional study characterizes the delivery of ambulatory surgical care for children across FASCs and HBOCs and tests for differences in patient characteristics and features of procedures being performed.

## Methods

We summarized known factors associated with the delivery of ambulatory surgical care at FASCs using a conceptual framework (eMethods 1 in Supplement 1) and followed the STROBE reporting guideline. Data sources included the Healthcare Cost and Utilization Project 2017 State Ambulatory Surgery and Services Databases from 3 states (Florida, New York, and Wisconsin) and Ambulatory Surgical Center Payment Rates (eMethods 2 in Supplement 1). We identified all patients aged 18 years or younger who underwent a common ambulatory surgical procedure (eTable 1 in Supplement 1). Patients with missing variables were excluded. Institutional review board approval and patient consent were waived because the study was determined to be nonhuman participant research.

The primary outcome was the odds of delivering ambulatory surgery at an FASC (vs an HBOC). Statistical analysis was performed from January 2021 through April 2023. Multiple logistic regression models with generalized estimating equations were used to examine associations between the independent variables and the outcome, while accounting for covariates and county-level clustered data. The key independent variable in the first model was payer status, categorized as private, public (Medicaid and CHIP), and other insurance status. In the second model, which was stratified by payer status, the key independent variable was an indicator for whether the surgery was expensive (defined based on procedure price; eFigure and eTable 2 in Supplement 1). Models controlled for age, sex, race and ethnicity, median household income, urban or rural location, chronic condition status, and states. We used SAS statistical software, version 9.4 (SAS Institute Inc), for statistical analyses. All *P* values were from 2-tailed tests, and results were deemed statistically significant at *P* < .05.

## Results

A total of 198 362 observations were identified from the 3 states in 2017, and 54 248 of the procedures (27.3%) were performed in an FASC. Differences in patient and procedure characteristics between FASCs and HBOCs were observed, except for age (Table 1). For example, the proportion of privately insured patients at FASCs and HBOCs was 55.8% (30 281 of 54 248) and 49.8% (71 746 of 144 114) (*P* < .001), respectively.

**Table 1.** Characteristics of Patients and Surgical Procedures

Characteristic	Freestanding (n = 54 248)	Hospital owned (n = 144 114)	*P* value
Age, median (IQR), y	7 (3-15)	7 (3-14)	.19
Sex			
Female	24 181 (44.6)	60 753 (42.2)	<.001
Male	30 067 (55.4)	83 361 (57.8)	
Race and ethnicity^a^			
Black	5328 (10.3)	17 067 (11.8)	<.001
Hispanic	10 798 (19.8)	22 174 (15.4)	
White	32 697 (60.3)	85 427 (59.3)	
Other	5425 (9.9)	19 446 (13.5)	
Payer status			
Private	30 281 (55.8)	71 746 (49.8)	<.001
Public (Medicaid and Children’s Health Insurance Program)	21 651 (39.9)	67 270 (46.7)	
Others	2316 (4.3)	5098 (3.5)	
Median household income national quartile for patient zip code			
Quartile 1 (lowest)	13 235 (24.4)	34 370 (23.9)	<.001
Quartile 2	15 425 (28.4)	41 369 (28.7)	
Quartile 3	14 468 (26.7)	37 309 (25.9)	
Quartile 4 (highest)	11 120 (20.5)	31 066 (21.6)	
Patient location			
Large metropolitan areas	35 324 (65.1)	87 885 (61.0)	<.001
Small metropolitan areas	15 519 (28.6)	40 466 (28.1)	
Micropolitan areas	2064 (3.8)	9688 (6.7)	
Not metropolitan or micropolitan	1341 (2.5)	6075 (4.2)	
No. of pediatric chronic conditions			
0	53 351 (98.4)	135 685 (94.2)	<.001
1	871 (1.6)	6809 (4.7)	
≥2	26 (0.1)	1620 (1.1)	
Price of surgical procedures^b^			
Expensive (≥90th percentile)	3946 (7.3)	9195 (6.4)	<.001
Moderate	50 302 (92.7)	134 919 (93.6)	
State			
Florida	35 642 (65.7)	52 010 (36.1)	<.001
New York	9996 (18.4)	68 370 (47.4)	
Wisconsin	8610 (15.9)	23 734 (16.5)	

^a^ Original race and ethnicity information is derived from patient discharge data and provided by Healthcare Cost and Utilization Project Partner organizations; the other category includes Asian or Pacific Islander, Native American, and other races.^6^

^b^ Price of procedures is bimodally distributed, and the top 80th, 85th, and 90th percentiles are all equal to $2039.50.

Results of the first model indicated that the odds of receiving an expensive procedure in an FASC for those with private insurance were almost 50% higher than those with public insurance (odds ratio [OR], 1.47; 95% CI, 1.26-1.71; *P* < .001) (Table 2). Patients who were Black (OR, 0.72; 95% CI, 0.58-0.90) or had 1 chronic condition (OR, 0.30; 95% CI, 0.21-0.42) had decreased odds of receiving surgery in an FASC. There were significant differences in the odds of receiving an expensive procedure in an FASC between patients covered by public and patients covered by private insurance. Specifically, among those privately insured, expensive procedures had higher odds of being performed in an FASC (OR, 1.37; 95% CI, 1.20-1.56). In contrast, among those who were enrolled in Medicaid and CHIP, expensive procedures had lower odds of being performed in an FASC (OR, 0.52; 95% CI, 0.34-0.79).

**Table 2.** Association of Patient and Procedure Characteristics with the Odds of Surgery Delivery at a Freestanding Ambulatory Surgical Center

Variable	Odds ratio (95% CI)		
	Model 1	Model 2 (private)	Model 2 (public)
Age	1.00 (0.99-1.01)	1.01 (0.99-1.02)	0.99 (0.98-1.00)
Sex			
Female	1.12 (1.08-1.16)	1.07 (1.04-1.11)	1.16 (1.09-1.24)
Male	1 [Reference]	1 [Reference]	1 [Reference]
Race and ethnicity			
Black	0.72 (0.58-0.90)	0.74 (0.62-0.88)	0.70 (0.54-0.91)
White	1 [Reference]	1 [Reference]	1 [Reference]
Hispanic	1.05 (0.86-1.28)	0.88 (0.74-1.03)	1.15 (0.90-1.48)
Other	1.20 (0.80-1.78)	1.43 (0.84-2.44)	1.00 (0.74-1.34)
Health insurance			
Private	1.47 (1.26-1.71)	NA	NA
Public	1 [Reference]	NA	NA
Other	1.18 (0.83-1.69)	NA	NA
Median household income national quartile for patient zip code			
Quartile 1 (lowest)	0.82 (0.65-1.04)	0.75 (0.59-0.96)	0.93 (0.71-1.22)
Quartile 2	0.82 (0.68-0.99)	0.85 (0.70-1.03)	0.89 (0.70-1.12)
Quartile 3	0.87 (0.73-1.02)	0.89 (0.75-1.05)	0.94 (0.75-1.19)
Quartile 4 (highest)	1 [Reference]	1 [Reference]	1 [Reference]
Patient location			
Large metropolitan	1 [Reference]	1 [Reference]	1 [Reference]
Small metropolitan	0.78 (0.54-1.12)	0.79 (0.56-1.11)	0.74 (0.45-1.22)
Micropolitan areas	0.69 (0.43-1.10)	0.74 (0.43-1.28)	0.60 (0.35-1.03)
Not metropolitan	0.57 (0.34-0.94)	0.60 (0.37-0.99)	0.49 (0.25-0.95)
No. of pediatric chronic conditions group conditions			
0	1 [Reference]	1 [Reference]	1 [Reference]
1	0.30 (0.21-0.42)	0.35 (0.26-0.46)	0.24 (0.15-0.39)
≥2	0.04 (0.02-0.06)	0.04 (0.02-0.08)	0.03 (0.01-0.09)
Price of surgical procedures			
Expensive	1 [Reference]	1.37 (1.20-1.56)	0.52 (0.34-0.79)
Moderate	0.89 (0.75-1.05)	1 [Reference]	1 [Reference]
State			
Florida	1 [Reference]	1 [Reference]	1 [Reference]
New York	0.18 (0.13-0.24)	0.17 (0.13-0.23)	0.15 (0.10-0.25)
Wisconsin	0.53 (0.34-0.83)	0.47 (0.30-0.75)	0.66 (0.41-1.06)

Abbreviation: NA, not applicable.

## Discussion

Results from this study suggest that FASCs may be selectively choosing more financially desirable, privately insured patients, while not treating those enrolled in Medicaid or CHIP. Results also indicated that, among those privately insured, FASCs may be selecting more expensive procedures, whereas among those enrolled in Medicaid and CHIP, FASCs are less likely to do so. Although geographic access to FASCs and HBOCs may account for some differences, we were unable to fully adjust for this factor due to a lack of information about their locations. Further studies are needed to explore geographic access to these facilities across diverse sociodemographic groups and examine whether the selective choice behavior leads to differences in patient outcomes and undermines cost savings.

## Notes

Supplement 1. **eMethods 1.** The Conceptual Framework Used in This Study
eReferences 1.
**eMethods 2.** Data Sources Used in This Study
eReferences 2.
**eTable 1.** A List of Common Ambulatory Surgical Procedures Included in This Study **eFigure.** The Price of Ambulatory Surgical Procedures **eTable 2.** A List of Expensive Ambulatory Surgical Procedures

Supplement 2.
Data Sharing Statement
