# Supplementary material for: Disparities in Delivery of Ambulatory Surgical Care for Children
Source: JAMA Netw Open. 2023 Jun 5;6(6):e2317018. doi: 10.1001/jamanetworkopen.2023.17018 (PMC10242420; doi:10.1001/jamanetworkopen.2023.17018)
Supplement: Supplement 1. — eMethods 1. The Conceptual Framework Used in This Study eReferences 1. eMethods 2. Data Sources Used in This Study eReferences 2. eTable 1. A List of Common Ambulatory Surgical Procedures Included in This Study eFigure. The Price of Ambulatory Surgical Procedures eTable 2. A List of Expensive Ambulatory Surgical Procedures [file jamanetwopen-e2317018-s001.pdf]

Supplementary Online Content

Tian Y, Allen LD, Ingram MCE, Raval MV. Disparities in delivery of ambulatory surgical care for children. *JAMA Netw Open.* 2023;6(6):e2317018. doi:10.1001/jamanetworkopen.2023.17018

eMethods 1. The Conceptual Framework Used in This Study
eReferences 1.
eMethods 2. Data Sources Used in This Study
eReferences 2.
eTable 1. A List of Common Ambulatory Surgical Procedures Included in This Study
eFigure. The Price of Ambulatory Surgical Procedures
eTable 2. A List of Expensive Ambulatory Surgical Procedures

This supplementary material has been provided by the authors to give readers additional information about their work.

© 2023 Tian Y et al. *JAMA Network Open.*

eMethods 1. The Conceptual Framework Used in This Study

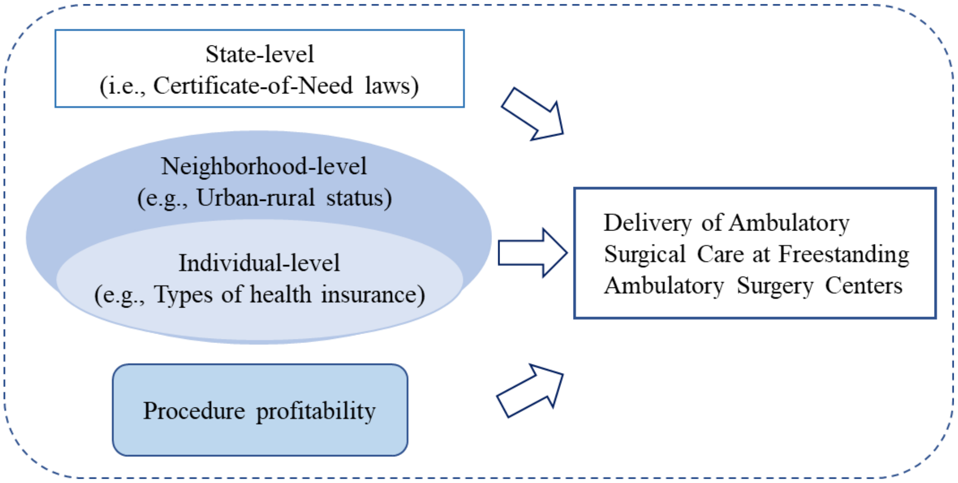

Recent literature on adult patient populations has identified various factors that are associated with the delivery of surgical care at Freestanding Ambulatory Surgery Centers (FASCs). These include Certificate-of-Need Laws, a state-level factor, which may delay the diffusion of FASCs,[1] neighborhood-level factors such as FASCs being more commonly located in affluent and urban areas with less competition for ambulatory surgical care,[2-3] and individual-level factors such as Black patients and patients with public health insurance being less likely to use FASCs.[4-5] In addition, studies have shown that the profitability of surgeries is positively associated with the likelihood of their delivery at FASCs.[6]

The primary outcome of this study was the odds of delivering ambulatory surgery at a FASC (versus a Hospital-Based Outpatient Center (HBOC)). Multiple logistic regression models with generalized equation estimation were used to examine associations between the independent variables and the outcome while accounting for covariates and county-level clustered data. Covariates included age, sex, race/ethnicity, median household income national quartile for patient ZIP code, urban-rural designation for the patient's county of residence, patient chronic condition status, and state (Table 2). The models also controlled for patient clustering within the same county.

eReferences 1.

1. Robertson, M. These 12 states have CON laws that don't restrict ASCs. Becker's ASC Review. Available at: https://www.beckersasc.com/benchmarking/these-12-states-have-con-laws-that-don-t-restrict-ascs.html Accessed on April 10, 2023.
2. Chatterjee, A., Amen, T. B., Khormaee, S. Trends in Geographic Disparities in Access to Ambulatory Surgery Centers in New York, 2010 to 2018. JAMA Health Forum. 2022 Oct 7;3(10):e223608.
3. Suskind A. M., Zhang, Y., Dunn, R. L., Hollingsworth J. M., Strope S. A., Hollenbeck B. K. Understanding the diffusion of ambulatory surgery centers. Surg Innov. 2015 Jun;22(3):257-65.
4. Janeway, M.G., Sanchez, S.E., Chen, Q., Nofal, M.R., Wang, N., Rosen, A., Dechert, T.A. Association of Race, Health Insurance Status, and Household Income With Location and Outcomes of Ambulatory Surgery Among Adult Patients in 2 US States. JAMA Surg. 2020 Dec 1;155(12):1123-1131.

© 2023 Tian Y et al. *JAMA Network Open.*

5. Strope S. A., Sarma, A., Ye, Z., Wei, J. T., Hollenbeck B. K. Disparities in the use of ambulatory surgical centers: a cross sectional study. BMC Health Serv Res. 2009 Jul 21;9:121.
6. Plotzke, M.R., Courtemanche, C. Does procedure profitability impact whether an outpatient surgery is performed at an ambulatory surgery center or hospital? Health Econ. 2011 Jul;20(7):817-30.

© 2023 Tian Y et al. *JAMA Network Open.*

eMethods 2. Data Sources Used in This Study

Data sources of this study included the Healthcare Cost and Utilization Project (HCUP) 2017 State Ambulatory Surgery and Services Databases (SASD) from three states (Florida, New York, and Wisconsin) and the 2017 Ambulatory Surgical Center (ASC) Approved Healthcare Common Procedure Coding System (HCPCS) Codes and Payment Rates.[1-2] The three states were selected as they submit an indicator to specify the ambulatory setting for discharge records and have a relatively large number of FASCs.[3] The ASC Approved HCPCS Codes and Payment Rates are published by the Centers for Medicare & Medicaid Services and provide eligible ambulatory procedures and their corresponding payment amounts.[2,4] This study used the 2017 Payment Rates data because Cost-to-Charge Ratio data is not available in HCUP SASD.[5]

eReferences 2.

1. SASD Database Documentation. Healthcare Cost and Utilization Project (HCUP). September 2021. Agency for Healthcare Research and Quality, Rockville, MD. Available at: www.hcup-us.ahrq.gov/db/state/sasddbdocumentation.jsp. Accessed on October 29, 2022.

2. Ambulatory Surgical Center (ASC) Approved HCPCS Codes and Payment Rates. Centers for Medicare & Medicaid Services. Available at: https://www.cms.gov/Medicare/Medicare-Fee-for-Service-Payment/ASCPayment/archive. Accessed on June 21, 2022

3. Introduction to the HCUP State Ambulatory Surgery and Services Databases (SASD). Healthcare Cost and Utilization Project (HCUP). June 2022. Agency for Healthcare Research and Quality, Rockville, MD. Available at: www.hcup-us.ahrq.gov/db/state/sasddist/SASD_Introduction.jsp. Accessed on April 10, 2023

4. Ambulatory Surgical Center Payment System. The Medicare Learning Network Booklet. Available at: https://www.hhs.gov/guidance/sites/default/files/hhs-guidance-documents/ambsurgctrfeepymtfctsht50809.pdf . Accessed on April 10, 2023

5. Cost-to-Charge Ratio (CCR) Files. Agency for Healthcare Research and Quality, Rockville, MD. Available at: https://hcup-us.ahrq.gov/db/ccr/costtocharge.jsp . Accessed on April 10, 2023

© 2023 Tian Y et al. *JAMA Network Open.*

eTable 1. A List of Common Ambulatory Surgical Procedures Included in This Study

The Clinical Classifications Software (CCS) categories of procedures listed below were used to identify surgical procedures that are frequently performed at both Hospital-Based Outpatient Centers (HBOCs) and Freestanding Ambulatory Surgery Centers (FASCs).

**CCS Category**	**CCS Category Description**
19	Other therapeutic procedures on eyelids, conjunctiva, cornea
21	Other extraocular muscle and orbit therapeutic procedures
22	Tympanoplasty
23	Myringotomy
30	Tonsillectomy and/or adenoidectomy
33	Other OR therapeutic procedures on nose, mouth and pharynx
70	Upper gastrointestinal endoscopy, biopsy
76	Colonoscopy and biopsy
115	Circumcision
118	Other OR therapeutic procedures, male genital
142	Partial excision bone
144	Treatment, facial fracture or dislocation
148	Other fracture and dislocation procedure
149	Arthroscopy
151	Excision of semilunar cartilage of knee
160	Other therapeutic procedures on muscles and tendons
161	Other OR therapeutic procedures on bone
162	Other OR therapeutic procedures on joints
170	Excision of skin lesion

The Clinical Classifications Software (CCS) for Services and Procedures is a software tool developed as a part of the Healthcare Cost and Utilization Project (HCUP) to classify Healthcare Common Procedure Coding System (HCPCS) codes into clinically meaningful categories. The list of common ambulatory surgical procedures is derived from the most frequently performed procedures at both HBOCs and FASCs.

© 2023 Tian Y et al. *JAMA Network Open.*

eFigure. The Price of Ambulatory Surgical Procedures

The most expensive procedures were determined based on the empirical distribution of the price data. As shown below, the price distribution is skewed. A bimodal distribution is observed in the price data, and the top 80th, 85th, 90th percentiles are all equal to $2039.5.

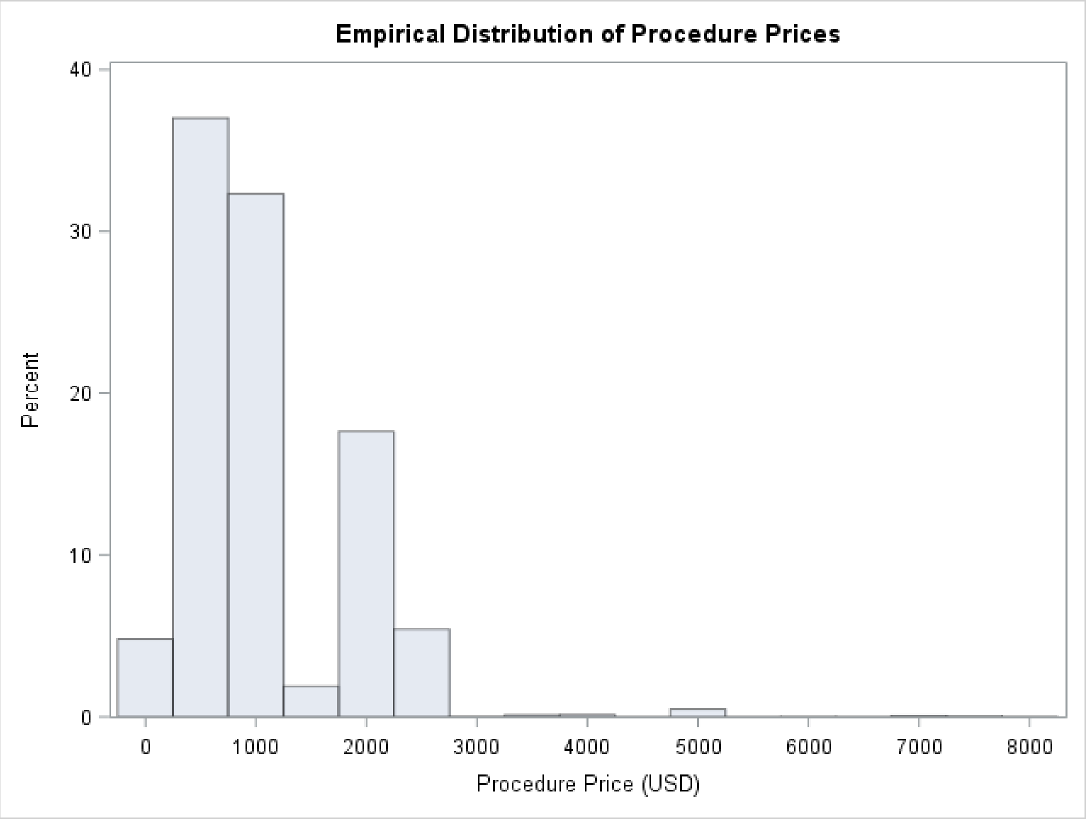

© 2023 Tian Y et al. *JAMA Network Open.*

eTable 2. A List of Expensive Ambulatory Surgical Procedures

**CCS Category**	**CCS Category Description**
148	Other fracture and dislocation procedure
149	Arthroscopy
161	Other OR therapeutic procedures on bone
162	Other OR therapeutic procedures on joints

The Clinical Classifications Software (CCS) for Services and Procedures is a software tool developed as a part of the Healthcare Cost and Utilization Project (HCUP) to classify Healthcare Common Procedure Coding System (HCPCS) codes into clinically meaningful categories.

© 2023 Tian Y et al. *JAMA Network Open.*
