# Supplementary material for: Disparities in Delivery of Ambulatory Surgical Care for Children
Source: JAMA Netw Open. 2023 Jun 5;6(6):e2317018. doi: 10.1001/jamanetworkopen.2023.17018 (PMC10242420; doi:10.1001/jamanetworkopen.2023.17018)
Supplement: Supplement 2. — Data Sharing Statement [file jamanetwopen-e2317018-s002.pdf]

Data Sharing Statement

Tian. Disparities in Delivery of Ambulatory Surgical Care for Children. *JAMA Netw Open*. Published June 05, 2023. doi:10.1001/jamanetworkopen.2023.17018

Data
Data available: No

Additional Information
Explanation for why data not available: Limitations for data source (AHRQ HCUP) user agreements
